# Dynamic Expression Profiles of Circular RNAs during Brown to White Adipose Tissue Transformation in Goats (*Capra hircus*)

**DOI:** 10.3390/ani11051351

**Published:** 2021-05-10

**Authors:** Xujia Zhang, Siyuan Zhan, Shizhong Yang, Tao Zhong, Jiazhong Guo, Jiaxue Cao, Yan Wang, Li Li, Hongping Zhang, Linjie Wang

**Affiliations:** 1 Farm Animal Genetic Resources Exploration and Innovation Key Laboratory of Sichuan Province, College of Animal Science and Technology, Sichuan Agricultural University, Chengdu 611130, China; zhangxujia@stu.sicau.edu.cn (X.Z.); siyuanzhan@sicau.edu.cn (S.Z.); zhongtao@sicau.edu.cn (T.Z.); jiazhong.guo@sicau.edu.cn (J.G.); jiaxuecao@sicau.edu.cn (J.C.); wangyan8108@sicau.edu.cn (Y.W.); lily@sicau.edu.cn (L.L.); zhp@sicau.edu.cn (H.Z.); 2 Institute of Liangshan Animal Husbandry and Veterinary Science, Xichang 615042, China; xcysz1565@163.com

**Keywords:** circRNA, brown adipose tissue, perirenal fat, goat

## Abstract

**Simple Summary:**

In our study, we launched RNA-seq in order to investigate the potential functions of circRNA during brown adipose tissue (BAT) to white adipose tissue (WAT) transformation. As a result, 6610 circRNAs and 61 differentially expressed circRNAs (DEcircRNAs) were identified. Moreover, 65 miRNAs were detected that could potentially interact with DEcircRNAs. The present study provides a detailed circRNA expression landscape and evidence for circRNA functions in the transformation from BAT to WAT.

**Abstract:**

Adipose tissues are mainly divided into brown adipose tissue (BAT) and white adipose tissue (WAT). WAT mainly functions to buffer excess calories, whereas BAT plays a role in the non-shivering thermogenesis to maintain body temperature and energy balance. Moreover, circRNAs play important roles in various biological processes. However, knowledge of the expression profile and function of circRNAs from BAT to WAT remains largely unknown. In this study, a total of 6610 unique circRNAs were identified in the perirenal adipose tissues of 1-day, 30-days, and 1-year goats. Functional annotation revealed that host genes of circRNAs were involved in some BAT-related pathways, such as the thyroid hormone signaling pathway, MAPK signaling pathway, and VEGF signaling pathway. Furthermore, a total of 61 DEcircRNAs were detected across three stages. Additionally, five selected circRNAs were validated by RNase R assay, qPCR, and Sanger sequencing. Finally, the circRNA–miRNA network was constructed between the DEcircRNAs and their miRNA binding sites.

## 1. Introduction 

Adipose tissue plays a critical role in regulating systemic metabolism and energy homeostasis through fatty acid and glucose metabolism [1,2,3]. It is known that adipose tissue can be generally divided into brown adipose tissue (BAT) and white adipose tissue (WAT). WAT functions to buffer surplus calories in the form of triacylglycerol (TG), which can release chemical energy when needed [4,5]. Different from WATs in morphology, function, and molecular level, BAT is a kind of thermogenic tissue specialized to dissipate chemical energy by uncoupling oxidative phosphorylation from ATP synthesis through uncoupling protein 1 (UCP1), an abundant mitochondrial protein distributed in inner membrane [6,7]. Recently, a kind of brown-like adipose tissue is obtained from WAT in response to chronic cold exposure and other stimulating conditions, such as food, exercise, drugs, and so on [8,9,10], which is called beige adipose tissue or “Brite” [11].

Circular RNA (circRNA) is a type of non-coding RNAs generally formed by special “back-splicing” reactions from pre-mRNA [12]. It is more stable than linear RNAs and resistant to digestion by RNase R because of its circular structure [13]. Increasing evidence have suggested that circRNAs can function as miRNA sponges and then regulate gene transcription by competing for miRNA binding. One of the most well-studied circRNAs is CDR1as, transformed from the antisense strand of *CDR1*, has over 70 miR-7 binding sites [14,15]. As a result, it notably inhibits insulin secretion in β cells of islet [16] and influences some neural diseases, such as synaptic dysregulation in neurocytes [17]. With the development of high throughput sequencing technology, more circRNAs have been recently reported in different cell types and tissues, such as the heart [18], brain [19], and muscle [20]. Recent studies have shown that circRNAs are abundant in adipose tissue and play critical regulatory role in lipogenesis. Liu et al. identified 6451 circRNAs in subcutaneous adipose tissue of pigs, revealing that circRNAs are dynamically expressed in adipocytes [21]. Arcinas et al. employed high-throughput sequencing and identified 6925 and 2380 circRNAs in human and mouse adipose tissues respectively. Furthermore, circTshz2-1 and circArhgap5-2 were subsequently demonstrated to be necessary for adipocytes differentiation [22]. Moreover, an exosomal circRNA, ciRS-133, has been verified to promote adipocyte differentiation and WAT browning via sponging miR-133 [23]. 

BAT plays an important role on the non-shivering thermogenesis through uncoupling protein (UCP1) [24]. The UCP1 enriched in inner membrane of mitochondria functions to uncoupling with ATPase, directly converting chemical energy into energy to maintain body temperature in cold environments [25]. There are large amounts of BAT at birth in larger mammals such as human and ruminants. Then, BAT is gradually replaced by WAT during postnatal life [26,27]. In a recent study, we found that there is an obvious transition from BAT to WAT after the birth of goats. The hematoxylin–eosin (HE) staining and *UCP1* gene expression analysis among three stages showed that the perirenal fat at 1 day (D1) was BAT, the expression of *UCP1* was at the highest level in D1, and then significantly decreased at 30 days (D30), reaching its lowest expression at 1 year (Y1) [28]. There was obvious transition from BAT to WAT during goat perirenal fat development from D1 to Y1. To investigate the dynamic circRNA expression profiles during BAT to WAT transformation, we chose D1, D30, and Y1 as our time point. In this study, we systematically identified and analyzed circRNAs expressions during the transformation from BAT to WAT. In addition, we performed Sanger sequencing and qPCR to validate the candidate circRNAs. We further found out the differentially expressed circRNAs (DEcircRNA) and then constructed DEcircRNA-miRNA interaction network to unveil the miRNA-sponge capabilities of circRNAs.

## 2. Materials and Methods

### 2.1. Animal and Sample Collection

For this study, 12 female Chuanzhong black goats at 1 day, 30 day, and 1 year after birth were raised at the breeding center of Sichuan Agricultural University, Ya’an, China (~1000 m altitude, 103.00° E, 29.98° N), annual temperature is 14 °C in average whereas humidity is 52%. In this study, Chuanzhong black goats were not related to each other. Moreover, we selected one of the twins from each doe. In addition, we chose four individuals with similar body weight at each stage (2.93 ± 0.27 kg, 6.72 ± 0.41 kg, and 30.98 ± 1.43 kg in D1, D30, and Y1, respectively). Furthermore, we calculated the carcass fat percentage; it showed a low intra-group variation (1.50 ± 0.18%, 5.25 ± 0.53%, and 12.39 ± 0.94% at three periods). Details of the carcass trait information was shown in Table S1. Does were fed with water ad libitum, and a standard diet (30% of concentrated feed contained) twice per day, at 07:00–09:00 a.m. and 04:00–06:00 p.m. All goats were fasted overnight and then injected intramuscularly by using su mian xin (xylazine hydrochloride, Shengda, Changchun, China) at a dose of 0.1 mL/kg *bw*. Under complete anesthesia, all goats were sacrificed by arterial bleeding. Then, the perirenal adipose tissues were collected and immediately frozen in liquid nitrogen and stored at −80 °C for RNA extraction and RNA sequencing.

### 2.2. RNA Extraction, Library Construction, and Sequencing

Total RNA of perirenal adipose tissues were isolated by TRIzol (Invitrogen, Carlsbad, CA, USA) according to manufacturer’s protocol. RNA concentration and purity were evaluated using NanoDrop 2000 spectrophotometer (Thermo Fisher Scientific, Waltham, MA, USA). The RNA integrality was assessed by RNA Nano 6000 Assay Kit of the Agilent 2100 Bioanalyzer (Agilent Technologies, Santa Clara, CA, USA). 

Then, twelve RNA-seq libraries were constructed. Briefly, 1.5 μg RNA were used for rRNA removal by the Ribo-Zero rRNA Removal Kit (Epicentre, Madison, WI, USA), followed by sequencing libraries construction using NEBNext^R^ Ultra^TM^ Directional RNA Library Prep Kit for Illumina^R^ (New England Biolabs, Beverly, MA, USA)). Then, Hiseq4000 platform (Illumina, San Diego, CA, USA) were launched for a 150 bp paired-end sequencing to get raw data, which could be obtained at the Sequence Read Archive (SRA) database (Accession no. PRJNA547456) (https://www.ncbi.nlm.nih.gov/bioproject/PRJNA547456, accessed on 15 October 2020).

### 2.3. Quality Control, Transcriptome Assembly, and circRNA Identification

Firstly, the raw reads were filtered to acquire clean reads by removing adapter- containing reads, poly-N-containing reads (over 5%) and low-quality reads. After that, all clean reads were aligned to goat reference genome [29] by BWA (v0.7.10) [30]. After that, reads were assembled using StringTie (v1.3.1) [31]. Mapped reads were used for the identification of circRNAs by CIRI2 (v2.0.5) [32] with default parameters to detect back-spliced reads (at least two unique back-spliced reads whereas obey the GU–AG rule). Differentially expressed circular RNAs (DEcircRNAs) among three stages were identified using the DESeq2 R package (v.1.30.0) [33] with false discovery rate (FDR) < 0.05 and |log 2 (fold change)| ≥ 1. In addition, reads were normalized by the algorithm of transcripts per million (TPM) [34]. For cluster analysis, heatmap was generated via pheatmap in R package, according to default parameters.

### 2.4. CircRNA Annotation

Gene Ontology (GO) and Kyoto Encyclopedia of Genes and Genomes (KEGG) were commonly used to help us understanding the potential functions of circRNAs. Linear transcripts were enriched according to their GO terms and visualized via topGO R package [35]. Besides, KOBAS (v2.0) [36] software were used to conduct the statistical enrichment of circRNAs host genes in the KEGG pathways.

### 2.5. Verification of circRNAs

RNase R digestion assay was launched according to the manufacture’s recommendation (Geneseed, Guangzhou, China). In detail, 5 μg of total RNA were incubated with 1U μg^−1^ RNase R or PBS for 10 min at 37 °C and 10 min at 70 °C. Then, treated RNAs were reverse transcribed to cDNA using PrimeScript^RT^ reagent Kit (Takara, Tokyo, Japan). After that, five candidate DEcircRNAs were randomly selected and circPrimer (v1.2) [37] were carried out to design divergent primers. Then, qPCR was used to quantify expression levels in different developmental stages using CFX96 connection (BIO-RAD, Hercules, CA, USA). Data were normalized with *TBP*, one of the most suitable reference genes according to our previous study [38]. Expression levels calculated via the 2^−ΔΔCt^ method.

### 2.6. CircRNA-miRNA Interaction

To detected target miRNAs, miRanda (v3.3a) [39], and RNA Hybrid (v2.1.1) [40] were launched with default parameters. Then, the circRNA–miRNA network was constructed according to the prediction of miRNA binding sites. Furthermore, we used Cytoscape (v3.3.0) [41] to describe the circRNA–miRNA interaction.

### 2.7. circRNA Conservation Analysis among Human, Mouse, and Goat

The analyses of circRNA conservation were conducted according to a previous study [42]. Mouse and human circRNA data were obtained from circBase (http://www.circbase.org, accessed on 24 April 2021), Basic Local Alignment Search Tool (BLAST, https://blast.ncbi.nlm.nih.gov/, accessed on 24 April 2021) was used for sequence alignment with a threshold of *e*-value < 1.0 × 10^−5^.

### 2.8. Statistical Analysis

Results were displayed as mean ± SEM; data were analyzed and visualized by GraphPad Prism 5. In addition, Student’s *t*-test was conducted for significant analysis. *p*-values less than 0.05 were considered significant, different letters revealed that genes expressed conspicuously different among each other (*p* < 0.05). 

## 3. Results

### 3.1. Characteristics of Goat Perirenal Fat in D1, D30, and Y1

**Figure 1:**
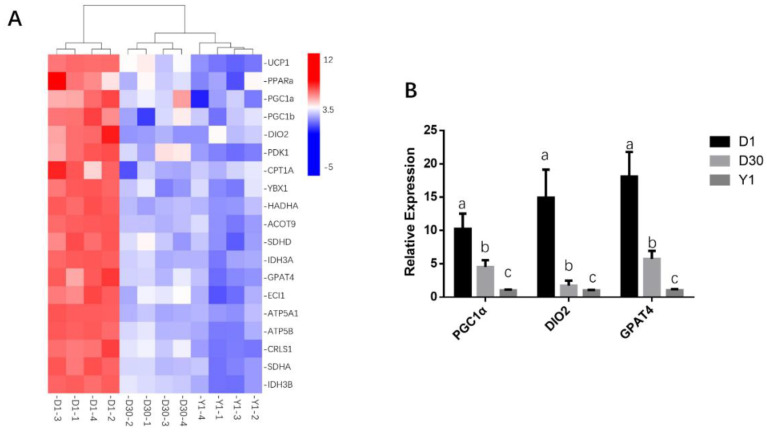
Characteristics of goat perirenal fat in 1 day (D1), 30 day (D30), and 1 year (Y1) (**A**) Heatmap clustering showed the BAT-related gene expression from RNA-seq. (**B**) qPCR verification showed the BAT-related marker gene expressions. *TBP* act as a negative control. Error bars represent standard error of mean (SEM), *n* = 4. The superscript in the histogram (a, b, c) showed the significance, different letters revealed that genes expressed conspicuously different among each other (*p* < 0.05).

To characterize the biological process of BAT development, we displayed a heatmap of 19 thermogenesis-related genes, such as *UCP1*, *DIO2*, *PPARα*, *PGC1α*, and *PGC1β* from our RNA-seq data. Cluster analysis showed that 12 samples were divided into three groups, D1 clustered to one group, whereas D30 and Y1 were divided into two separate clusters. Moreover, as expected, those BAT-related marker genes were showed the highest expression in D1 and showed a constantly downregulated from D30 to Y1 (Figure 1A). In addition, we launched qPCR to detect some traditional BAT marker genes, such as *DIO2*, *PGC1α*, and *GPAT4* (Figure 1B). Consistent with the heatmap mentioned above, all of those BAT-marker genes showed high expressions in D1, then downregulated in D30 and expressed lowest in Y1. Those results indicated that perirenal adipose tissue in D1, D30, and Y1 have their unique gene expression profiles.

### 3.2. Identification and Characteristics of circRNAs in Goat Perirenal Fat

**Figure 2:**
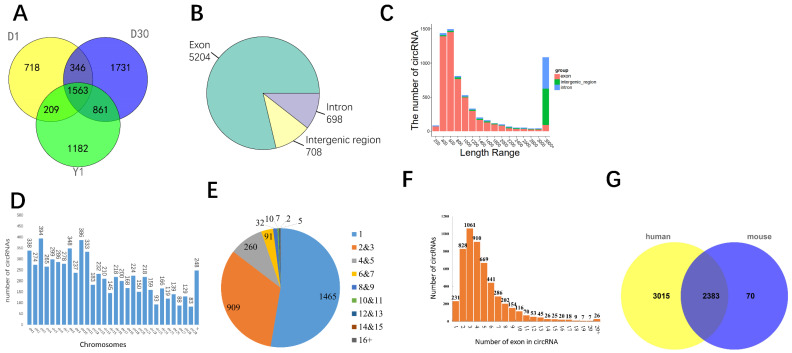
Identification and characteristics of Circular RNA in goat perirenal fat. (**A**) The number of circRNAs in three stages. (**B**) Distribution of circRNAs in the genome. The green region represents exon region, the yellow represents intergenic region, and the purple represents intron region. (**C**) circRNA length distribution of all circRNAs from twelve tissues. (**D**) The number of circRNAs in each goat chromosomes. (**E**) circRNA isoforms produced from one gene. (**F**) Exon number consisted in one circRNA. (**G**) circRNA conservation analysis among goat, human, and mouse. The yellow circle showed the population of conserved human circRNAs, whereas the blue circle showed the conserved mouse circRNAs. The junction part demonstrated the conserved circRNA number among goat, human, and mouse.

Here, a total of 1,429,821,402 reads were obtained from 12 total RNA libraries at three stages. Then we used CIRI2 software to detect head-to-tail junction reads (at least two unique back-spliced reads). In total, we found 396,252 paired junction reads and 6610 unique circRNAs (Table S2). Among these identified circRNAs, 2836, 4501, and 3815 were detected in D1, D30, and Y1, respectively. Additionally, 1563 circRNAs were found in all three stages (Figure 2A). According to origination analysis, the majority of circRNAs (5204, 78.7%) were originated from exons, whereas 708 (10.7%) were consisted of intergenic and 698 (10.6%) from intron (Figure 2B). We further analyzed the length of circRNAs, the result illuminated that the length range of 200 to 1000 nt contains 4270 circRNAs (64.60%) (Figure 2C).

To characterize the origin of circRNAs, we analyzed the host gene’s location of circRNAs. It demonstrated that circRNAs were widely distributed in all chromosomes. In addition, chromosome 3 produced the most circRNAs (394), whereas chromosome 29 contains the least for only 83 circRNAs (Figure 2D). In addition, 1465 (52.68%) genes generated only one circular RNA, while two or three isoforms were produced from 909 (32.69%) genes, the others yield three or more circular isoforms (Figure 2E). Of all 5204 exonic circRNAs, we found that most of them were formed by multiple exons and only 231 circRNAs consisted of one exon, which takes 4.44% of all exonic circRNAs (Figure 2F). Finally, we launched conservation analysis of circRNAs. As a result, 5398 (81.7%) and 2453 (37.1%) circRNAs were conserved with human and mouse respectively, thereinto, 2383 (36.0%) circRNAs were simultaneously conserved among goat, human, and mouse (Figure 2G). These results provided a comprehensive catalog of circRNAs in goat white and brown adipose tissues.

### 3.3. Function Analysis of circRNA

**Figure 3:**
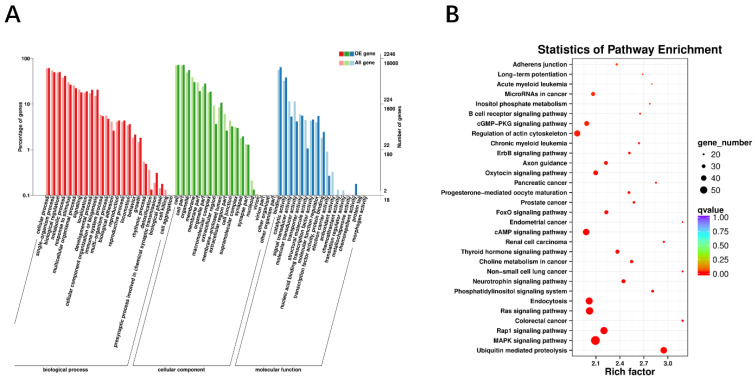
Functions analysis of circRNA. (**A**) Gene Ontology (GO) analysis of host genes. (**B**) KEGG enrichment analysis shows top 30 significantly enriched KEGG pathways for circRNA host genes.

To further explore the putative functional categories of circRNAs, GO and KEGG analysis for their host genes were conducted. For GO analysis, genes were organized into hierarchical categories to discover their regulatory networks based on three domains. For biological process, terms were mostly enriched in cellular process, single-organism process, response to stimulus, biological regulation, metabolic process, and multicellular organismal process. For cellular component, terms were enriched in cell, organelle, cell part, and organelle part. Moreover, molecular function terms were enriched in binding, catalytic activity, and transporter activity (Figure 3A). Subsequently, KEGG enrichment analysis indicated that all host genes were significantly enriched in 36 signaling pathways (Table S3). Among them, terms were most significantly enriched in ubiquitin-mediated proteolysis, endocytosis, MAPK signaling pathway, Rap1 signaling pathway, and Ras signaling pathway. Besides, we also found that genes were clustered into thyroid hormone signaling pathway, FoxO signaling pathway, Wnt signaling pathways and VEGF signaling pathway (Figure 3B), revealing that some enriched pathways might related to adipose tissue development and energy metabolism.

### 3.4. Identification and Function Analysis of Differentially Expressed circRNAs (DE circRNAs) in Goat Perirenal Fat

**Figure 4:**
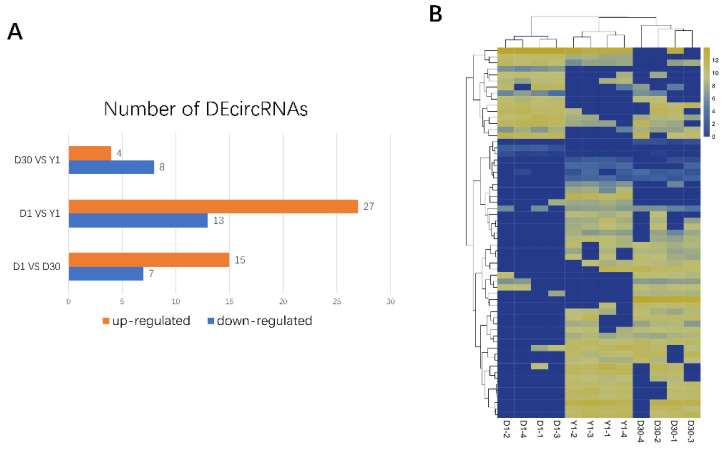
Identification and function analysis of DEcircRNAs in goat perirenal fat. (**A**) Numbers of DEcircRNAs in three stages of perirenal fat, the differences were compared to the latter group. (**B**) Heatmap clustering shows all DEcircRNAs in three different stages.

To evaluate the expressions of circRNAs, reads count was normalized by TPM and DEcircRNAs were filtrated with the standard of FDR < 0.05 and |log 2 (fold change)| ≥ 1. A total of 61 DEcircRNAs were identified during three developmental stages (Table S4). 15 upregulated and 7 downregulated circRNAs were observed in D1 compared to D30, 27 upregulated and 13 downregulated circRNAs were identified in D1 compared to Y1, 4 upregulated and 8 downregulated circRNAs were detected in D30 compared to Y1 (Figure 4A). In order to determine the relationships among three stages, clustering analysis of all DEcircRNAs was performed. Heatmap revealed that three stage groups showed their specific clusters, D1 formed one cluster whereas D30 and Y1 formed another two clusters (Figure 4B). Taken together, these results showed that the differentially expressed circRNAs in D1, D30, and Y1 may have specific functions during brown to white adipose tissue transformation in goats. 

### 3.5. Validation of circRNAs

**Figure 5:**
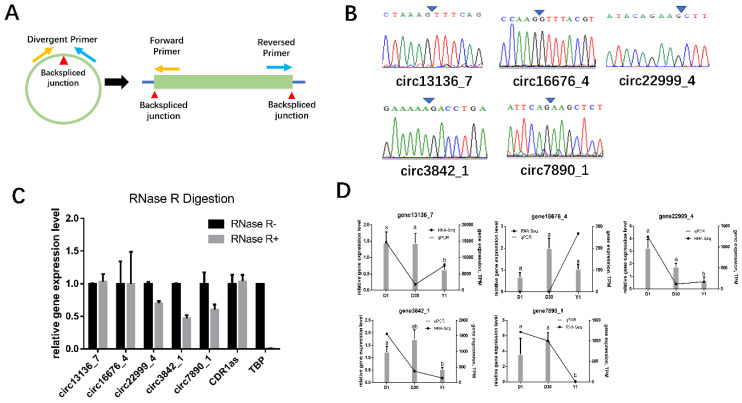
Validation of circRNAs. (**A**) Schematic representation of divergent primer. Orange arrows represent the forward primer whereas the blue shows reversed primer, the red triangle point to the back spliced junction. (**B**) Sanger sequencing shows head-to-tail junctions. Blue triangle shows back spliced junctions. (**C**) RNase R digestion assay. TBP was acted as negative control whereas CDR1as acted as positive control. Data were normalized according to the value measured in the mock treatment. (**D**) Validation of five DEcircRNA expressions via qPCR. Column shows qPCR results whereas RNA-seq data are shown as polyline. Error bars represent standard error of mean (SEM), *n* = 4. The superscript in the histogram showed the significance, different letters revealed that genes expressed conspicuously different among each other (*p* < 0.05).

To confirm the circRNAs generated from RNA-seq data, five candidates (circ7890_1, circ3842_1, circ16676_4, circ13136_7, circ22999_4) were randomly selected for experimentally validating using divergent primers (Table S5 and Figure 5A). Furthermore, PCR products were detected by Sanger sequencing to confirm back-spliced junctions (Figure 5B). To eliminate the possibility of duplicated genes or exon reshuffling, RNase R assay was launched to verify those candidates according to the endonuclease tolerance of circRNA (Figure 5C). Moreover, we chose CDR1as, one of the most famous circRNAs, as our positive control whereas *TBP* as negative control. After RNase R treatment, qPCR was conducted to detect relative gene expressions. As a result, *TBP* was efficiently depleted whereas those candidate circRNAs were still enriched after RNase R treatment. Subsequently, qPCR was used to examine their expressions at different stages, as shown in Figure 5D, the qPCR expression tendency was consistent with the RNA-seq data. These results indicated that the pipeline for identifying putative circRNAs and the RNA-seq results were reliable. 

### 3.6. Putative Functions of DEcircRNAs Act as miRNA Sponges

**Figure 6:**
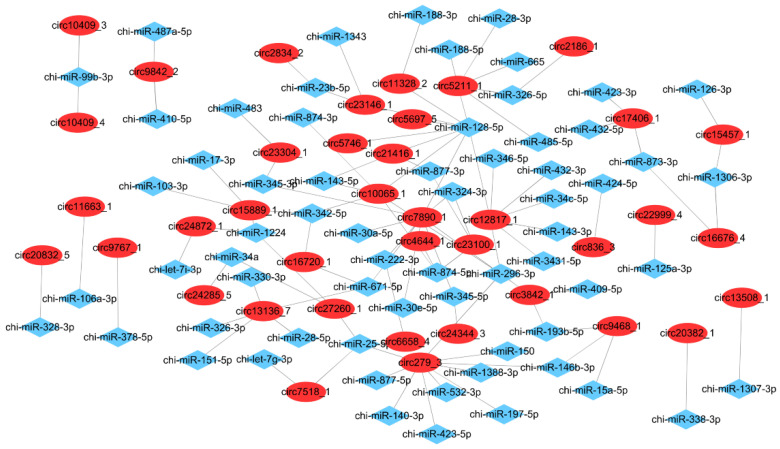
Target miRNA detection of DEcircRNAs. Red ellipses represent DEcircRNAs and blue rhombuses point to miRNA.

We constructed DEcircRNA-miRNA interaction network using RNA Hybrid and miRanda to unveil the miRNA-sponge capabilities of circRNAs (Figure 6). As a result, we found 65 target miRNAs (Table S6). Notably, we identified several brown adipose related miRNAs from this interactive network such as chi-miR-328, chi-miR-146-3p, chi-miR34a, chi-miR-150, and chi-miR-30 family. In addition, most circRNAs could sponge no more than five miRNAs; however, two circRNA (circ10065_1 and circ5211) could sponge six miRNAs, whereas circRNA circ12817_1 could interact with seven miRNAs. Besides, circ7890_1 could sponge nine miRNAs, and circ279_3 could sponge the most, up to 10 miRNAs. Interestingly, we found that chi-miR-128-5p could sponge nine circRNAs, and chi-miR-296-3p had five target circRNAs. The result revealed that circRNA might interact with miRNAs to regulate BAT development via acting as a molecular sponge.

## 4. Discussion

In large mammals, such as humans and ruminants, BAT is recruited at birth and UCP1 enriched in mitochondria initiates the non-shivering thermogenesis to adapt the cold challenge of the extra-uterine environment [43,44]. Therefore, elucidating the molecular mechanism of brown adipogenesis in ruminants is important for the ability to sustain central temperature in the postnatal environmental conditions. CircRNAs act as regulators of gene expression and are involved in many physiological and metabolism processes [45]. However, the differential expression profiles of circRNA during the transformation from BAT to WAT have not been extensively studied. In this study, we identified 6610 circRNAs among 12 perirenal fat tissues (D1, D30, and Y1). This provides a valuable resource to study the regulatory function of circRNA in brown adipose tissue. 

The 6610 unique circRNAs were mapped in 2779 genes. Previous studies showed that the functions of circRNAs were associated with its host gene and could regulate the expression level of their host gene [46]. For example, ACC1 can generate its circular transcription rather than liner gene with the help of c-Jun in the conditions of serum deprivation [47]. In our study, KEGG analysis showed that host genes were enriched in some BAT-related terms, such as thyroid hormone, MAPK, and VEGF signaling pathway. Research demonstrated that thyroid hormone is a critical hormone to adaptive and obligatory thermogenesis in order to cope with cold stimulation. Mechanically, it could uncouple electron transport to regulate mitochondrial biogenesis, active brown adipose tissue, and induce browning of WAT [48,49,50]. It is known that MAPK signaling pathway regulates adipocyte differentiation [51]. Research revealed that cardiac natriuretic peptides and cryptotanshinone could regulate mitochondrial biogenesis and thermogenesis in BAT [52,53]. Moreover, research verified that VEGF knock down and anti-VEGF antibody injection both impair the capacity of beige adipose tissue biosynthesis. Moreover, in BAT, transgene of VEGF enhances UCP1 and PGC1α expression and increases thermogenesis during cold exposure [54,55,56]. These results suggested that circRNAs may be involved in some biological processes during the transformation from BAT to WAT.

In addition, a total of 61 DEcircRNAs were detected among three stages. Some brown adipose tissue-enriched circRNAs interested us because their host genes have a potential function related to brown adipogenesis. For example, circ20832_5 is derived from neuregulin 4 (*NRG4*), which is a BAT-secreted adipokine. Recent research showed that neuregulin 4 secreted from BAT could directly activate BAT or induce the browning of WAT through paracrine [57,58]. Circ3842_1 is derived from AMP-activated protein kinase γ2 (*PRKAG2*) gene, which were involved in AMPK signaling pathway. *PRKAG2* is an important member of the AMPK gamma subunit. As is known to us all, AMPK is a heterotrimeric protein that play a multifaceted role in cellular energy metabolism [59]. AMPK α1 and α2 is essential for mitochondrial integrity and regulates thermogenesis in adipose tissue [60,61]. Moreover, circ7890_1 is derived from folliculin-interacting protein 1 (*FNIP1*). Previous studies reported that folliculin (*FLCN*), the binding protein of *FNIP1*, could regulate the browning of WAT, and suppress mitochondrial biogenesis [62,63,64]. In addition, circ16676_4 is derived from Acetyl-CoA carboxylase 2 (*ACC2*). There is a higher rate of lipolysis and fatty acid oxidation in the adipocytes of ACC2−/− mutant mice compared with wild type mice [65]. Moreover, circACC1 was proven to increase glycolysis and β-oxidation, so circ16676_4 might influence BAT fat transforming. Moreover, a previous study has proved that circNrxn2 regulated WAT browning [66]. In this study, we found neurexin 1 (Nrxn1) could generate circular RNA and was highly expressed in D1 and D30, indicating that circNrxn1 (circ11663_1) might have a regulatory function in BAT development. These findings indicated that differential expression of circRNAs may be involved in the transformation from BAT to WAT and provided us with some valuable clues about the functions of circRNAs.

Moreover, circRNAs play important roles on transcription regulation through the way of ceRNA mechanism [14]. In this study, we constructed a DEcircRNA–miRNA interaction network and a total of 65 miRNAs were predicted to target with DEcircRNAs. Interestingly, we identified several brown adipose related miRNAs from this interactive network. For example, circ279_3, derived from Spindle and centriole-associated protein 1 gene (*Spice1*), could sponge chi-miR-140-3p, chi-miR-146b-3p, and chi-miR-150, which play important roles in adipogenesis and thermogenesis. Among them, miR-146b-3p and miR-150 were reported to be involved in WAT browning process, whilst miR-140-3p could positively regulate WAT adipogenesis [67,68,69]. Moreover, the circular transcript of *FNIP1*, circ7890_1, could sponge miR-30 family (miR-30a end miR-30e). MiR-30a could inhibit adipocyte differentiations [70] and miR-30e promote adipocyte differentiation by targeting lipoprotein receptor-related protein 6 gene [71]. MiR-30b/c promote thermogenesis and adipogenesis of beige fat [72]. Circ3842_1 (circulated from *PRKAG2*), the target of chi-miR-193b-5p, was expressed with the highest level in D1. A previous study reported that miR-193b is a key regulator of brown fat development and could induce myoblasts to differentiate into brown adipocytes [73]. Circ24285_5 and circ27260_1 (circulated from the dedicator of cytokinesis protein 1 and AF4/FMR2-2) could sponge chi-miR-34a, which inhibits brown fat formation by suppressing the browning activators FGF21 and SIRT1 [74]. These results suggested that some circRNAs may participate in the transformation from BAT to WAT by the ceRNA mechanism. However, their regulatory mechanisms of circRNA and their target miRNAs still need to be investigated. 

## 5. Conclusions

We provided a comprehensive catalog of circRNAs in the transformation from BAT to WAT. Furthermore, we built a circRNA–miRNA regulatory network to reveal the miRNA-sponge capabilities of circRNAs. Our dataset serves as an important resource to study the regulatory function of circRNAs during the transformation from BAT to WAT.

## Data Availability

The data presented in this study are available on request from the corresponding author.

## Supplementary Materials

The following are available online at https://www.mdpi.com/article/10.3390/ani11051351/s1, Table S1. Comparison of carcass traits among three stages. Table S2: The information of all identified circRNAs. Table S3: KEGG analysis to host genes of all identified circRNAs. Table S4: List of differently expressed circRNAs from three developmental stages in perirenal fat. Table S5: Sequences of qPCR primers for circRNAs and negative control. Table S6: Identification of target miRNAs to DEcircRNAs.
